# Gli2, hedgehog and TCR signalling

**DOI:** 10.18632/oncotarget.5017

**Published:** 2015-07-26

**Authors:** Anna L. Furmanski, Tessa Crompton

**Affiliations:** Asthma UK Fellow, Department of Life Sciences, University of Bedfordshire, Luton, UK

**Keywords:** Immunology and Microbiology Section, Immune response, Immunity

A structurally and functionally diverse T-cell repertoire is essential for each of the key roles of the adaptive immune system: elimination of foreign pathogens, provision of immunological memory and the maintenance of tolerance to self. T-cells develop in the thymus and then migrate to the periphery where they undergo activation and functional differentiation following contact with cognate antigen via their T-cell receptor (TCR). TCR signaling and co-stimulation trigger a cascade of intracellular events that culminate in the activation of AP-1, NFκB and NFAT proteins, which mediate the subsequent transcriptional response. Throughout their lifespan T-cells make multiple fate decisions. During development and activation, the strength, context and timing of the TCR signal is important in determining the functional activity of the cell. CD4+ T-helper (Th) cells in particular exhibit context-dependent functional plasticity, which is apparently regulated by a complex network of cytokine signals, many of which are derived from the local cell milieu.

Given the potential plasticity of CD4+ T-cells and the growing appreciation for the role of a cell's local environment in its fate and function, it is important to understand the ways in which environmental cues integrate with TCR signaling. Such signals include molecules that activate Gli transcription factors in T-cells. Gli proteins are the downstream effectors of canonical Hedgehog (Hh) signalling. There is an established negative regulatory role for Hh signalling and/or the activation of Gli-dependent transcription at the TCR-dependent stages of T-cell development in the thymus [1–4]. Hh/Gli-driven signaling is also important in the differentiation and function of peripheral Th2 cells, which are involved in asthma and allergic immune responses [5] and in naïve CD4+ T-cells during TCR signaling [1, 2, 6]. Gli transcription factors are expressed in wild-type (WT) T-cells [6, 7]. Our recent study showed that expression of the transcriptional activator form of Gli2 (Gli2A) in CD4+ T-cells decreased the ability of T-cells to activate, proliferate and produce interleukin-2 (IL-2) in response to TCR stimulation. T-cell calcium flux in response to TCR ligation was also impaired in Gli2A cells, which also showed lower expression of nuclear NFAT2 [6]. Microarray analysis of transcriptional responses to Gli2A and Gli2R (the repressor form of Gli2) in CD4+ T-cells revealed a wide range of differentially expressed transcripts including members of the wider morphogen family and other genes involved in differentiation and death. Importantly, genes encoding key TCR signalling molecules were differentially expressed between WT, Gli2A and/or Gli2R CD4+ cells. These included components of the AP-1 transcriptional complex (*Jun, Fos, Fosb*) and members of the NF-kappa-B signaling pathway (*Ikbkb*). DNA binding activities of AP-1 and NFκB were diminished in activated Gli2A T-cells, whereas Gli2R T-cells showed enhanced binding of NFκB to DNA compared to WT T-cells [6]. Together our data show that Gli2-mediated transcription in T-cells modulates TCR signalling and T-cell activation.

**Figure 1:**
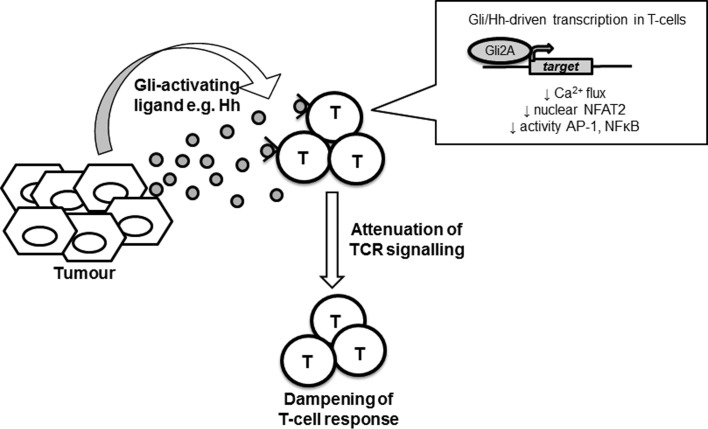
Proposed immunomodulatory role for Hh/Gli signaling Schematic diagram suggesting a potential role of Hh/Gli signaling in anti-tumour immunity. Certain tumour cells secrete Hh protein ligands, activating Gli-dependent transcription in tumour-infiltrating T-cells. Gli2A attenuates the intracellular events downstream of TCR signaling including the calcium flux and AP-1/NFκB activity. The subsequent transcriptional response to the TCR stimulus is dampened, skewing the T-cell response and potentially aiding tumour evasion.

These observations link previous findings that Gli2A both alters T-cell repertoire selection [1, 3] and CD4+ Th differentiation [5], as modulation of both of these processes could be explained by ‘dampened’ TCR signaling. There are wide implications for these observations. Gli-activating Hh proteins may be upregulated in tissues during damage, repair or chronic inflammation. This raises the intriguing possibility that Hh and/or other Gli-activating ligands could act as novel immunomodulators when released by tissue. In evolutionary terms, a tissue-derived ligand that ‘down-tunes’ TCR signals to favour Th2 differentiation would be useful if the tissue were infected with a parasite. It is also possible that Hh/Gli signaling between tissue and immune cells acts as a balance between acute inflammatory and immune resolution repair phases. Interestingly, several cancers, particularly of epithelial origin, express Hh ligands. The dampening of TCR signal transduction by Hh/Gli signaling to local T-cells could therefore be an unexplored mechanism of tumour immune evasion (Figure 1). Hh pathway inhibitors are used therapeutically in some cancers, thus there may be additional hidden benefits to the use of these drugs in tumours that secrete Hh ligands.

In summary, activation of Gli-dependent transcription in CD4+ T-cells acts to modulate TCR signals. The plastic nature of T-helper cells means that they are particularly susceptible to microenvironmental signals such as those potentially delivered by Gli-activating ligands.
